# Contraceptive dynamics during COVID-19 in sub-Saharan Africa: longitudinal evidence from Burkina Faso and Kenya

**DOI:** 10.1136/bmjsrh-2020-200944

**Published:** 2021-02-12

**Authors:** Celia Karp, Shannon N Wood, Georges Guiella, Peter Gichangi, Suzanne O Bell, Philip Anglewicz, Elizabeth Larson, Linnea Zimmerman, Caroline Moreau

**Affiliations:** 1 Department of Population, Family and Reproductive Health, Johns Hopkins University Bloomberg School of Public Health, Baltimore, Maryland, USA; 2 Institut Supérieur des Sciences de la Population (ISSP)/University of Ouagadougou, Ouagadougou, Centre, Burkina Faso; 3 International Centre for Reproductive Health Kenya (ICRH-K), Mombasa, Kenya; 4 Soins et Santé Primaire, CESP Centre for Research in Epidemiology and Population Health U1018 INSERM, Villejuif, France

**Keywords:** contraception behavior, COVID-19, epidemiology, family planning services, reproductive health services, surveys and questionnaires

## Abstract

**Introduction:**

Evidence from health emergencies suggests COVID-19 will disrupt women’s sexual and reproductive health (SRH). In sub-Saharan Africa, which experiences the highest rates of unintended pregnancy and unsafe abortion globally, COVID-19 is projected to slow recent progress toward universal access to contraceptive services.

**Methods:**

We used longitudinal data collected from women at risk of unintended pregnancy in Burkina Faso (n=1186) and Kenya (n=2784) before (November 2019–February 2020) and during (May–July 2020) COVID-19 to quantify contraceptive dynamics during COVID-19; examine sociodemographic factors and COVID-19 experiences related to contraceptive dynamics; and assess COVID-19-related reasons for contraceptive non-use. Bivariate and multivariate logistic regressions were used to examine correlates of contraceptive dynamics amid COVID-19.

**Results:**

Most women did not change their contraceptive status during COVID-19 (68.6% in Burkina Faso and 81.6% in Kenya) and those who did were more likely to adopt a method (25.4% and 13.1%, respectively) than to discontinue (6.0% and 5.3%, respectively). Most women who switched contraceptives were using methods as or more effective than their pre-pandemic contraception. Economic instability related to COVID-19 was associated with increased contraceptive protection in Burkina Faso but not in Kenya. Altogether, 14.4% of non-contraceptive users in Kenya and 3.8% in Burkina Faso identified COVID-19-related reasons for non-use.

**Conclusions:**

The vast majority of women at risk of unintended pregnancy did not change their contraceptive status during COVID-19, and more women adopted than discontinued methods. A minority of women reported COVID-19-related reasons for non-use, underscoring the importance of expanding safe modes of service delivery during health crises.

**Key messages**

- Most women at risk of unintended pregnancy in Burkina Faso and Kenya did not change contraceptive use status between the pre-COVID-19 (November 2019–February 2020) and COVID-19 (May–July 2020) periods.
- Women who changed contraceptive use status were more likely to adopt than discontinue contraception; those who switched transitioned to as or more effective methods relative to pre-COVID-19.
- Fourteen percent of women at risk of unintended pregnancy and not using contraception in Kenya and 4% in Burkina Faso reported a COVID-19-related reason for non-use.

## Introduction

The COVID-19 pandemic reached sub-Saharan Africa (SSA) in February 2020; as of mid-September, it had caused nearly 33 000 deaths.^1 2^ While the direct impact of COVID-19 in SSA is not as widespread as in other regions,^1^ the indirect costs are potentially greater. Public health measures to reduce infections have destabilised economies and weakened fragile healthcare systems.^3^ Consequences are expected to be particularly devastating for women’s sexual and reproductive health (SRH) given mobility restrictions, service disruptions and economic insecurity, which inhibit access to time-sensitive care.^4–9^ Adding to the existing challenges of satisfying women’s need for contraception in the region,^10^ the pandemic may increase rates of unintended pregnancy and unsafe abortion.^11 12^ Women’s fertility intentions and need for contraception may also change due to unpredictable economic circumstances.^13^

Contraceptive discontinuation is a growing contributor to unmet need for contraception^14^ and is expected to increase amid COVID-19-induced service closures and contraceptive stockouts.^12^ Small-scale, facility-based studies in SSA have documented disruptions to contraceptive services,^15 16^ yet there are limited data to quantify how such changes affect contraceptive behaviours.

In this study we describe contraceptive dynamics during COVID-19 in two SSA countries, Burkina Faso and Kenya. These countries are at distinct stages of development, reflected in differences in total fertility rates (TFR) (5.19 Burkina Faso vs 3.49 Kenya)^17^ and modern contraception prevalence rates (mCPR) (30.7% Burkina Faso vs 56.4% Kenya).^18^ In both countries, responses to COVID-19, including social distancing, restricted gatherings and closed borders, were implemented in March 2020. We aimed to assess levels and correlates of women’s individual contraceptive use dynamics during COVID-19 and understand COVID-19-related reasons associated with contraceptive non-use.

## Methods

### Study overview

This analysis uses Performance Monitoring for Action (PMA) data from two national cohorts of women in Burkina Faso and Kenya. Data were collected pre-COVID-19, via face-to-face interview, from November 2019 to February 2020 (Burkina Faso: n=6590; Kenya: n=9477) and during COVID-19, via phone interview, from May to July 2020 (Burkina Faso: n=3518; Kenya: n=5972). Pre-COVID-19 participants were selected using PMA’s multiple-stage sampling approach, starting with randomly selecting census enumeration areas (EAs), stratified by region in Kenya and rural/urban residence in Burkina Faso, and selecting 35–40 households per EA. We included all women aged 15–49 years from selected households. Women provided consent to be recontacted for subsequent interviews and those who owned phones (70.2% of women in Burkina Faso and 67.7% in Kenya) were eligible to participate in the COVID-19 survey; response rates were >99% among successfully recontacted women in both countries. The pre-COVID-19 survey collected information on women’s sociodemographic characteristics and contraceptive behaviours. The COVID-19 survey focused on health behaviours and COVID-19 impacts (online supplemental file S1). Both surveys received approval from in-country ethical committees, including the Comité d’Ethique Institutionnel Pour La Recherche en Santé (Burkina Faso - No. A14-2020) and Kenyatta National Hospital-University of Nairobi Ethics Research Committee (Kenya - No. P241/04/2020), and the COVID-19 survey received approval from the Johns Hopkins Bloomberg School of Public Health (IRB No. 12407).

10.1136/bmjsrh-2020-200944.supp1 Supplementary data

### Analytical sample

We limited analysis to women at potential risk of unintended pregnancy before and during COVID-19. Women were considered ‘at risk’ if they ever had sexual intercourse, were not pregnant or trying to give birth in the next year, and did not report sterility. We further restricted the sample to women who were in-union pre-COVID-19 (as a proxy for sexual activity), as information about sexual activity was not collected in the COVID-19 survey. The final analytical sample included 1186 women in Burkina Faso and 2784 women in Kenya.

### Patient and public involvement

Women from the community were not directly involved in formulating research questions nor interpretation of the results. However, they consented to share their experiences, providing the data for this study. A national advisory group, including members of the ministry of health, health providers, and civil society groups, were consulted about country-specific priorities and results interpretation.

### Measures

Our primary outcome was *contraceptive use dynamics* between the pre-COVID-19 and COVID-19 surveys, distinguishing four categories: (1) consistent non-use, (2) consistent use, (3) adoption (change from non-use to use) and (4) discontinuation (change from use to non-use).

We constructed additional secondary outcome measures, including *contraceptive adoption* (binary—among non-users pre-COVID-19), *discontinuation* (binary—among users pre-COVID-19) and *switching* (no change in effectiveness, change to less effective, change to more effective—among users during both timepoints). Method effectiveness was assessed via three categories: *highly effective long-acting* (intrauterine device, implant or sterilisation), *effective short-acting* (injectables, pills or diaphragm) and *less effective short-acting* (condoms, other barrier/traditional methods, emergency contraception, standard days or lactational amenorrhea method). Additionally, we asked women *reasons for contraceptive non-use* with response options focused on COVID-19-related factors, including health facility closures, fear of infection during care, restricted mobility, or contraceptive stockouts.

We considered a number of sociodemographic, reproductive health and COVID-19-related factors linked to contraceptive behaviours. Sociodemographic factors comprised age, parity, education, wealth and residence. Reproductive health factors included emotional response to potential pregnancy during COVID-19, assessed by asking women their reaction if they learnt they were pregnant at the time of the survey, and intention to use contraception among non-users pre-COVID-19. We combined pre-COVID-19 contraceptive use and contraceptive intentions into four categories: (1) non-user with no intentions to use, (2) non-user with intentions to use, (3) user of short-acting methods and (4) user of long-acting methods. COVID-19-related circumstances included concerns about infection, loss of household income due to COVID-19, household food insecurity since COVID-19, ability to socially distance, and concerns about future income loss due to COVID-19.

### Analytical methods

Descriptive statistics examined contraceptive use dynamics during COVID-19; bivariate analysis assessed factors associated with these changes. Multivariate logistic regressions examined: (1) contraceptive adoption among non-users pre-COVID-19, (2) discontinuation among users pre-COVID-19, and (3) switching among users before and during COVID-19. The switching analysis examined factors related to shifts to more effective methods, among women who were not using highly effective long-acting methods pre-COVID-19, and shifts to less effective methods, among women using highly effective or effective short-acting methods pre-COVID-19. Finally, we described the proportion of non-contraceptive users who reported COVID-19-related reasons for non-use. All analyses were stratified by country. Multicollinearity was assessed by examining correlations between covariates and variance inflation factors (VIFs). As correlations and VIFs did not exceed 0.6 and 2.0, respectively, all covariates were retained in multivariate analysis. Complete case analysis was utilised, as missingness of covariates was less than 1%.

Analyses were conducted in STATA 16 (College Station, TX, USA); weighting accounted for complex survey design, non-response, phone ownership, and differential COVID-19 survey loss to follow-up.

## Results

Sample characteristics are presented in online supplemental table S1. About 15.2% of women in Burkina Faso lived in urban areas compared with 27.0% in Kenya. On average, women were 30.3 and 32.7 years old in Burkina Faso and Kenya, and 50.7% and 43.3% had four or more children, respectively. Some 73.3% of Burkinabe women had never attended school, while 39.9% completed secondary education or higher in Kenya. Three-quarters of women in Burkina Faso and 92.6% in Kenya suffered household income loss due to COVID-19, and 12.3% and 23.2% of women, respectively, reported increased food insecurity during COVID-19. Approximately, three-quarters of women in both countries were very concerned about contracting the virus. Pre-pandemic, two-thirds of Burkinabe women were not using contraception; 18.1% used a long-acting method and 15.0% relied on short-acting contraception. In Kenya, 74.4% of women were using contraception (37.6% and 36.8% used short- and long-acting methods, respectively) pre-COVID-19.

10.1136/bmjsrh-2020-200944.supp2 Supplementary data

**Figure 1:**
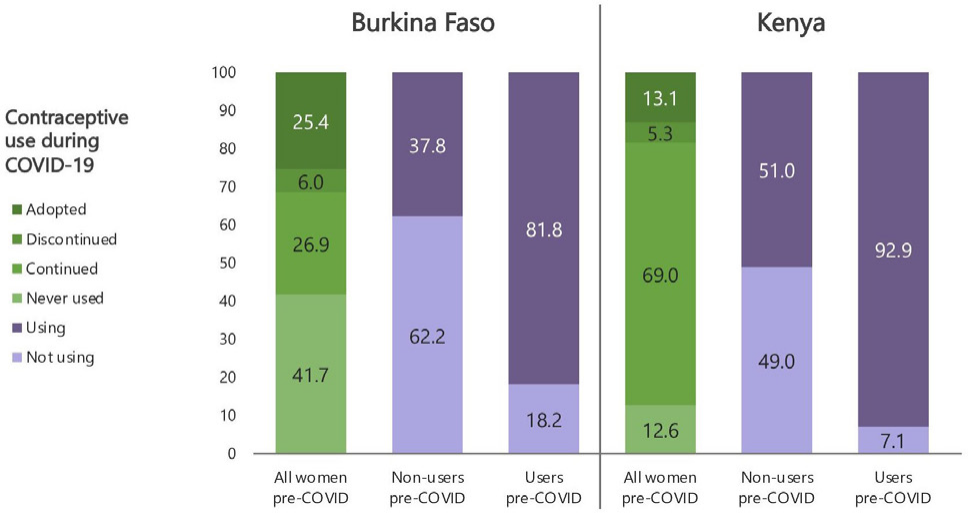
Changes to contraceptive use status between pre-COVID-19 and during COVID-19 by country.

**Figure 2:**
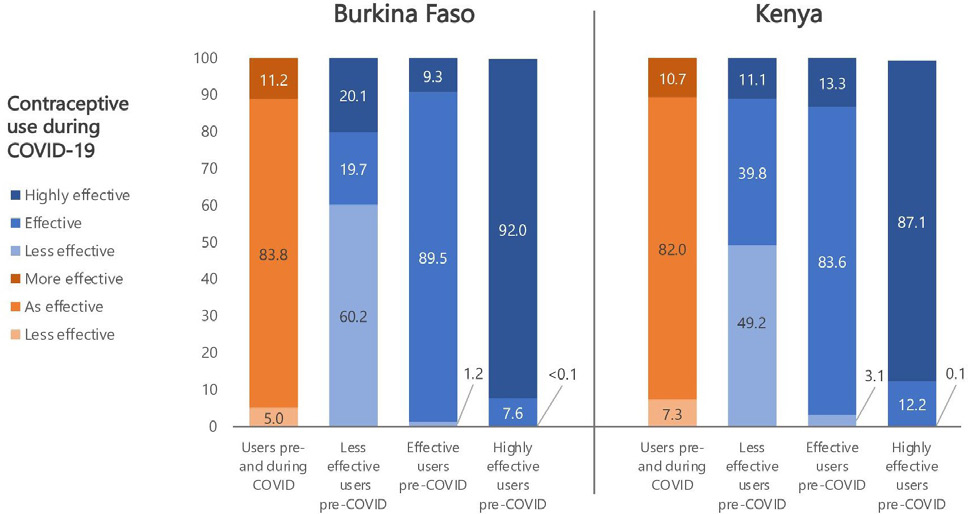
Change in women’s individual contraceptive practices and method type by country and pre-COVID-19 contraceptive use.

Altogether, 26.9% (95% CI 22.3 to 32.1) of women in Burkina Faso and 69.0% (95% CI 66.5 to 71.4) in Kenya remained users during COVID-19, while 41.7% (95% CI 34.9 to 48.9) and 12.6% (95% CI 10.8 to 14.5), respectively, remained non-users (figure 1). In both countries, more women adopted contraception (25.4% in Burkina Faso (95% CI 20.4 to 31.1) and 13.1% in Kenya (95% CI 11.4 to 13.1)) than discontinued (6.0% (95% CI 3.7 to 9.5) and 5.3% (95% CI 4.4 to 6.5), respectively). Among consistent users, about 10% switched to more effective methods (81.6% and 70.6% of whom switched to long-acting methods in Burkina Faso and Kenya, respectively), while fewer shifted to less effective methods (5.0% in Burkina Faso and 7.3% in Kenya; figure 2).

Distributions of women’s changes in contraceptive use and results of multivariate models for contraceptive adoption/discontinuation are presented in online supplemental table S2 and table 1. Kenyan women who were very concerned about COVID-19 were more likely to change contraceptive status, with greater odds of adoption and discontinuation. In addition, concerns about COVID-19’s impact on future economic prospects in Kenya were associated with greater discontinuation. Burkinabe women who were very concerned about COVID-19 were less likely to adopt a method. In contrast, Burkinabe women who experienced partial income loss or heightened food insecurity during COVID-19 were more likely to adopt contraception and were also less likely to discontinue.

**Table 1:** Factors associated with contraceptive discontinuation and adoption among users and non-users pre-COVID, by country

Factor	Burkina Faso						Kenya					
	Discontinuation among users at baseline (n=673)			Adoption among non-users at baseline (n=512)			Discontinuation among users at baseline (n=2078)			Adoption among non-users at baseline (n=706)		
	AOR	95% CI		AOR	95% CI		AOR	95% CI		AOR	95% CI	
Sociodemographic												
Residence												
Urban	ref			ref			ref			ref		
Rural	1.55	0.55	4.32	0.71	0.37	1.39	1.13	0.70	1.83	0.68	0.41	1.13
Age (years)												
25–34	ref			ref			ref			ref		
15–19	**0.19**	**0.05**	**0.66**	0.59	0.24	1.48	0.47	0.22	1.02	1.63	0.71	3.76
35–49	2.18	0.89	5.33	1.60	0.77	3.31	1.07	0.64	1.80	0.87	0.53	1.42
Education*												
Lower	ref			ref			ref			ref		
Higher	**0.35**	**0.15**	**0.81**	1.05	0.57	1.94	1.03	0.69	1.52	**1.64**	**1.05**	**2.56**
Wealth												
Low	ref			ref			ref			ref		
Middle	0.75	0.16	3.43	0.52	0.25	1.07	1.25	0.74	2.11	0.93	0.56	1.53
High	0.35	0.10	1.24	0.49	0.19	1.23	0.97	0.53	1.77	0.85	0.47	1.54
Reproductive health												
Parity (births)												
0–1	ref			ref			ref			ref		
2–3	0.64	0.21	1.97	1.22	0.46	3.19	0.35	0.17	0.73	2.14	1.01	4.52
3+	0.34	0.09	1.24	0.73	0.16	3.33	0.32	0.12	0.85	1.39	0.58	3.35
Emotional response to potential pregnancy												
Very happy	ref			ref			ref			ref		
Happy	2.65	0.43	16.23	2.50	1.20	5.20	0.94	0.47	1.85	**2.56**	**1.22**	**5.40**
Neutral	0.77	0.08	7.19	0.66	0.25	1.74	0.77	0.39	1.55	**4.69**	**1.91**	**11.52**
A little unhappy	2.78	0.49	15.86	0.43	0.11	1.71	1.21	0.60	2.45	**3.82**	**1.73**	**8.44**
Very unhappy	1.40	0.25	7.82	1.38	0.56	3.42	1.05	0.58	1.90	**4.99**	**2.42**	**10.29**
Contraceptive use and intentions at baseline												
Non-use, no intent				ref						ref		
Non-use with intent				2.63	1.42	4.90				**4.91**	**3.11**	**7.74**
Use of short-acting	ref						ref					
Use of long-acting	**0.30**	**0.14**	**0.65**				0.27	0.17	0.43			
COVID-19-related												
Household income loss												
None	ref			ref			ref			ref		
Partial	0.96	0.36	2.57	2.43	1.24	4.76	1.88	0.68	5.24	0.91	0.35	2.34
Complete	1.88	0.59	6.02	1.31	0.65	2.64	2.38	0.80	7.04	1.11	0.43	2.85
Concern about future loss of income due to COVID-19												
No	ref			ref			ref			ref		
Yes	1.16	0.28	4.81	1.31	0.37	4.67	**8.06**	**1.06**	**61.41**	1.11	0.48	2.59
Food insecurity during COVID-19												
None	ref			ref			ref			ref		
Chronic stable	3.64	0.64	20.78	1.20	0.51	2.81	0.61	0.28	1.32	1.03	0.55	1.93
Increased	**0.11**	**0.02**	**0.76**	3.35	1.24	9.07	1.11	0.68	1.81	0.89	0.53	1.48
Able to socially distance												
No	ref			ref			ref			ref		
Yes	1.09	0.32	3.73	0.79	0.30	2.08	1.21	0.80	1.83	1.37	0.85	2.21
Concerned about becoming infected with COVID-19												
Concerned	ref			ref			ref			ref		
Very concerned	2.16	0.94	4.97	0.46	0.22	0.96	0.85	0.49	1.49	**1.85**	**1.03**	**3.32**
A little/not concerned	**8.98**	**1.97**	**41.02**	0.38	0.11	1.29	1.61	0.72	3.59	1.05	0.40	2.75

*Education in Burkina Faso distinguished women who never went to school (lower) from women who went to school (higher). In Kenya, education distinguished women who attended secondary school or higher (higher) from those who had less education (lower).

†Multivariate regression adjusted for all other covariates listed and accounted for the complex survey design. Values in bold type indicate p<0.05.

AOR, adjusted odds ratio; CI, confidence interval; ref, reference.

Contraceptive use dynamics also varied by sociodemographic characteristics and pregnancy acceptability (table 1). In Kenya, more educated, higher parity women, and those who expressed unhappiness related to potential pregnancy, were more likely to adopt contraception. Women with higher education were also less likely to discontinue use. These relationships were not observed in Burkina Faso, where younger women were the only subsample less likely to discontinue contraception.

Among contraceptive non-users, 3.8% and 14.4% of women in Burkina Faso and Kenya, respectively, reported pandemic-related reasons for non-use (online supplemental table S3). Fear of infection at health facilities was the most frequently reported COVID-19-related reason, associated with 1.9% and 9.5% of non-use in Burkina Faso and Kenya, respectively.

Multivariate results for contraceptive switching are presented in table 2. Burkinabe women were more likely to shift to more effective contraception if they experienced COVID-19-related impacts, such as partial household income loss, yet were less likely to do so if they were very concerned about potential infection. In Kenya, women who experienced sustained food insecurity before and during COVID-19 were less likely to switch to more effective methods, relative to women reporting stable food resources.

**Table 2:** Multivariate correlates and predictors of switching to more and less effective methods during COVID-19 among women at risk of unintended pregnancy, by country

Factor	Burkina Faso						Kenya					
	More effective method*			Less effective method†			More effective method*			Less effective method†		
	(n=300)			(n=508)			(n=977)			(n=1765)		
	AOR	95% CI		AOR	95% CI		AOR	95% CI		AOR	95% CI	
Sociodemographic factors												
Residence												
Urban	ref			ref			ref			ref		
Rural	1.22	0.34	4.32	0.89	0.24	3.34	1.19	0.73	1.94	1.13	0.64	2.01
Age (years)												
25–34	ref			ref			ref			ref		
15–19	1.42	0.39	5.09	1.36	0.35	5.29	0.86	0.43	1.72	0.90	0.44	1.84
35–49	**0.24**	**0.07**	**0.81**	2.34	0.80	6.83	0.90	0.58	1.41	0.98	0.63	1.52
Education												
Lower	ref			ref			ref			ref		
Higher	2.37	0.82	6.83	1.76	0.53	5.93	1.16	0.72	1.87	0.75	0.50	1.13
Wealth												
Low	ref			ref			ref			ref		
Middle	**13.18**	**2.02**	**85.85**	**13.4**	**1.83**	**98.25**	0.65	0.40	1.05	0.94	0.57	1.54
High	**9.65**	**1.83**	**50.78**	5.25	0.80	34.49	0.74	0.42	1.31	0.85	0.47	1.53
Reproductive health												
Parity (births)												
0–1	ref			ref			ref			ref		
2–3	3.49	0.69	17.65	5.51	0.70	43.4	0.69	0.34	1.43	0.58	0.33	1.05
4+	**8.74**	**1.43**	**53.24**	1.89	0.26	13.5	0.78	0.34	1.80	**0.49**	**0.25**	**0.96**
Emotional response to potential pregnancy												
Very happy	ref			ref			ref			ref		
Happy	2.63	0.49	14.14	3.10	0.43	22.3	0.89	0.4	1.96	0.84	0.37	1.91
Mixed feelings	0.39	0.08	2.00	1.48	0.25	8.76	1.30	0.71	2.37	1.09	0.55	2.19
A little unhappy	0.41	0.06	2.72	2.41	0.57	10.19	0.86	0.39	1.87	0.92	0.43	1.95
Very unhappy	1.47	0.27	8.01	2.16	0.48	9.67	1.21	0.60	2.46	1.32	0.68	2.54
COVID-19-related factors												
Household income loss												
None	ref			ref			ref			ref		
Partial	**4.73**	**1.36**	**16.46**	1.57	0.40	6.12	0.89	0.44	1.80	1.46	0.67	3.18
Complete	2.42	0.44	13.18	2.81	0.59	13.40	0.90	0.46	1.74	1.01	0.48	2.12
Concern about future loss of income due to COVID-19												
No	ref			ref			ref			ref		
Yes	1.87	0.54	6.50	1.50	0.32	6.95	1.15	0.43	3.13	1.26	0.50	3.14
Food insecurity during COVID-19‡												
None							ref			ref		
Chronic stable							**0.34**	**0.12**	**0.94**	**1.30**	0.71	2.38
Increased							0.99	0.61	1.60	0.74	0.43	1.28
Able to socially distance												
No	ref			ref			ref			ref		
Yes	2.56	0.85	7.76	2.27	0.92	5.64	1.34	0.82	2.18	1.13	0.75	1.71
Concerned about becoming infected with COVID-19												
Concerned	ref			ref			ref			ref		
Very concerned	**0.17**	**0.06**	**0.47**	1.00	0.34	2.93	1.25	0.66	2.38	0.98	0.55	1.74
A little/not concerned	0.51	0.13	1.99	1.48	0.41	5.29	1.53	0.47	5.05	0.67	0.19	2.34

Multivariate regression adjusted for all other covariates listed and account for the complex survey design. Values in bold type indicate p<0.05.

*Among women not using highly effective contraception at baseline.

†Among women using effective/highly effective contraception at baseline.

‡Sample sizes for food insecurity were too small in Burkina Faso to detect differences among women who used contraception at baseline and follow-up.

AOR, adjusted odds ratio;CI, confidence interval; ref, reference.

## Discussion

This analysis of two national cohorts of women at risk of unintended pregnancy in Burkina Faso and Kenya who were interviewed before and during COVID-19 finds a complex relationship between the pandemic and contraceptive use. Most women did not change contraceptive status in the early months of COVID-19, and those who did were more likely to adopt contraception than to discontinue. Few women switched methods during COVID-19, most of whom shifted to more effective, long-acting contraception. Contraceptive dynamics related to concerns about COVID-19’s economic impact, although associations varied between countries. Additionally, 4–14% of non-users identified COVID-19-related barriers to contraceptive use.

Concerns about becoming infected with COVID-19 were associated with lower adoption and less switching to more effective methods among Burkinabe women. Fear of contracting COVID-19 was invoked by 2% and 10% of non-users as a reason for non-use in Burkina Faso and Kenya, respectively. These results align with studies reporting an indirect impact of epidemics on SRH service utilisation.^19 20^ Public health messaging during crises must be clear and consistent, balancing information about health threats with guidance on how people should seek care.

The relationship between economic-related impacts of COVID-19 and contraceptive dynamics differed across contexts, underscoring the complicated and context-dependent effect of economic shocks on fertility intentions and contraceptive behaviours. In Burkina Faso, economic instability in the form of household income loss or food insecurity was associated with greater contraceptive protection—through adoption, continuation, and switching to more effective methods. In contrast, Kenyan women who were concerned about their economic prospects were more likely to discontinue contraception, and those experiencing food insecurity were less likely to switch to more effective methods. Prior research suggests women adapt childbearing decisions when economic circumstances deteriorate, some adopting contraception to delay childbearing, others accelerating childbearing plans to strengthen social and economic support.^13 21^ While our analysis was restricted to women who wanted to avert childbearing in the next year, more than one-quarter indicated they would be happy if they learnt they were pregnant; in Kenya, women’s pregnancy acceptability was inversely associated with contraceptive adoption. Providers should consider how women’s perspectives towards pregnancy may change in the context of health and economic crises to tailor counselling that meets women’s evolving needs.

Greater contraceptive adoption, relative to discontinuation, during the early stage of COVID-19 underscores the resilience of women and health systems in these contexts. While nearly two-thirds of women reported difficulties accessing health services, the vast majority (87%) were successful in accessing care. Women’s ability to navigate health systems is reflected in the 26% and 13% of women in Burkina Faso and Kenya, respectively, who adopted contraception. Moreover, one in five women switched methods during COVID-19, mostly transitioning to methods more or equally effective than their pre-COVID-19 method. These results align with findings from early stages of the Zika epidemic in Brazil during which women often adopted or switched to more effective contraception.^22^ Safe mobile outreach programmes, which extend SRH services beyond facility walls, may increase contraceptive reach during public health emergencies.

While these findings suggest limited consequences of service disruptions on contraceptive use, results should be interpretated with caution. The transition from face-to-face to telephone surveys, required during COVID-19, resulted in 47% attrition in Burkina Faso. A study in Burkina Faso found a 14 percentage point higher contraceptive prevalence in a telephone survey compared with a population-based survey conducted face-to-face; half the difference was attributable to telephone ownership^22^ while the other half remained unexplained.^23 24^ Our weighted analysis addresses the potential bias related to telephone ownership but does not correct for differential responses by survey mode. Further, this study was conducted early in the pandemic and may not capture long-term effects of COVID-19 restrictions, as facilities may have had sufficient contraceptive commodities to operate relatively unaffected in the first months of COVID-19, yet face stockouts over time. In addition, contraceptive adoption or shifts to more effective methods may have occurred prior to the pandemic—between the time of the two surveys—especially in Burkina Faso, where a 2019 healthcare strike disrupted SRH services. The effects of COVID-19 are likely intertwined with other ongoing shocks to health, economic and social systems, which also shape contraceptive decisions and services.

Our findings may overestimate COVID-19’s role in contraceptive non-use, as response options were read aloud; however, given the low proportion of women identifying a COVID-19-related reason for non-use, this overestimation would reinforce our conclusions of COVID-19’s limited impact on contraceptive practices early in the pandemic. We also proxied women’s risk of unintended pregnancy based on marital status due to a lack of information about sexual activity during COVID-19; a more encompassing definition would include all sexually active women who wanted to avoid pregnancy. Finally, our sample size did not enable assessment of potential interactions among sociodemographic characteristics and COVID-19 experiences and resulted in large confidence intervals for some factors.

Despite these limitations, this study strengthens understanding of contraceptive dynamics early in the pandemic, accounting for women’s COVID-19 perceptions and concerns. Improved understanding of women’s changing contraceptive behaviours in response to COVID-19 disruptions can inform future public health preparedness and response efforts to ensure a continuity of essential SRH services. Communication efforts should centre on transparency around COVID-19 safety protocols to maximise reach and promote women’s comfort in accessing care.

## Conclusions

This prospective study demonstrates that most contraceptive users in Burkina Faso and Kenya sustained use, and more women adopted than discontinued contraception, early in the pandemic. Economic impacts and COVID-19 concerns were associated with contraceptive use dynamics, and 4% of non-users in Burkina Faso and 14% in Kenya identified COVID-19-related barriers to use. Safe modes of contraceptive service delivery during health emergencies and counselling that directly addresses women’s potential concerns as a result of the pandemic, especially their fears of facility-based infection, are central to ensuring continuity of quality SRH care amid public health crises.

## Data Availability

Data are available in a public, open access repository. Individual, deidentified participant data that underlie the results reported in this article will be publicly accessible to researchers by January 2021.
