# Supplementary material for: Contraceptive dynamics during COVID-19 in sub-Saharan Africa: longitudinal evidence from Burkina Faso and Kenya
Source: BMJ Sex Reprod Health. 2021 Feb 12;47(4):252–60. doi: 10.1136/bmjsrh-2020-200944 (PMC7886665; doi:10.1136/bmjsrh-2020-200944)
Supplement: Supplementary data [file bmjsrh-2020-200944supp002.pdf]

Supplemental Table S1. Characteristics of respondents by country

	**Burkina Faso** (N=1,186)		**Kenya** (N=2,784)	
	n	%	n	%
Sociodemographic				
**Residence**				
Urban	808	*15.2*	964	*27.0*
Rural	378	*84.8*	1,820	*73.0*
**Age**				
15-24	236	*31.5*	390	*18.9*
25-34	514	*38.5*	1,220	*40.8*
35-49	436	*30.0*	1,174	*40.2*
**Parity**				
0-1	191	*16.1*	356	*13.4*
2-3	475	*33.2*	1,242	*43.3*
4+	520	*50.7*	1,186	*43.3*
**Education***				
Lower	530	*73.3*	1,530	*60.1*
Higher	654	*26.7*	1,254	*39.9*
**Wealth**				
Low	134	*36.6*	822	*38.4*
Middle	229	*40.1*	1,084	*34.5*
High	823	*23.4*	878	*27.1*
Reproductive health				
**Contraceptive use and intentions before COVID-19**				
Not using with no intention	193	*23.6*	342	*12.2*
Not using but intends to use	320	*43.2*	364	*13.4*
Using short-acting method	358	*15.0*	1094	*37.6*
Using long-acting method	315	*18.1*	984	*36.8*
**Contraceptive use during COVID-19**				
No	385	*47.7*	489	*17.9*
Yes	799	*52.3*	2295	*82.1*
COVID-19-related				
**Economic loss due to COVID-19**				
None	263	*23.1*	175	*6.3*
Partial	741	*61.0*	1451	*50.8*
Complete	182	*15.9*	1157	*42.8*
**Worried about future income loss due to COVID-19**				
No	137	*10.5*	98	*3.9*
Yes	1,045	*89.5*	2684	*96.1*
**Food insecurity during COVID-19**				
None	1,031	*79.3*	1990	*68.4*
Chronic stable	58	*8.5*	208	*8.4*
Increased	96	*12.3*	586	*23.2*
**Able to socially distance**				
No	404	*25.0*	834	*31.2*
Yes	781	*75.0*	1950	*68.8*
**Concerned about becoming infected with COVID-19**				
Very concerned	759	*73.9*	2238	*79.1*
Concerned	242	*15.1*	361	*13.0*
A little/not concerned	184	*11.1*	184	*8.0*

**Education in Burkina Faso distinguished women who never went to school (lower) from women who went to school (higher). In Kenya, education distinguished women who have secondary school or higher (higher) from those who have less education (lower). Unweighted totals (n) and proportions (%).*

Karp C, *et al*. *BMJ Sex Reprod Health* 2021; 47:252–260. doi: 10.1136/bmjsrh-2020-200944

Supplemental Table S2. Contraceptive use dynamics during COVID-19 relative to pre-COVID-19 contraceptive use status by sociodemographic and COVID-19-related characteristics and site

	Burkina Faso					Kenya				
	Non-use	Continue	Discontinue	Adopt	p-value	Non-use	Continue	Discontinue	Adopt	p-value
Overall contraceptive status	41.7	26.9	6.0	25.4		12.6	69.0	5.3	13.1	
Baseline characteristics										
**Residence**										
Urban	21.2	56.5	7.8	14.6	**<0.001**	6.7	75.9	5.9	11.6	**<0.001**
Rural	45.4	21.6	5.7	27.3		14.8	66.5	5.1	13.6	
**Age**										
15-24	50.0	22.4	2.0	25.5	0.090	5.6	74.4	4.5	15.6	**<0.001**
25-34	38.6	30.1	7.6	23.7		8.5	74.0	6.0	11.5	
35-49	37.0	27.5	8.0	27.4		20.0	61.5	5.0	13.5	
**Parity**										
0-1	45.3	30.4	2.4	21.9	0.150	8.7	69.1	8.4	13.9	**<0.001**
2-3	46.0	23.7	5.6	24.8		7.8	73.8	5.1	13.3	
4+	37.8	27.9	7.4	27.0		18.5	64.3	4.5	12.6	
**Education**[a]										
Lower	45.3	20.2	6.5	28.0	**<0.001**	15.5	67.3	4.6	12.7	**<0.001**
Higher	32.1	45.0	4.5	18.4		8.2	71.7	6.4	13.7	
**Wealth**										
Low	38.1	20.3	7.0	34.5	**<0.001**	16.0	65.1	4.1	14.9	**0.004**
Middle	51.2	22.4	4.5	21.9		11.9	69.7	6.2	12.2	
High	31.3	44.8	6.9	17.0		8.6	73.8	6.0	11.6	
Reproductive health										
**Contraceptive use and intentions**										
Not using with no intention	73.6	-	-	26.4	n/a	69.6	-	-	30.4	n/a
Not using but intends to use	55.9	-	-	44.1		30.3	-	-	69.7	
Using short-acting method	-	75.0	25.0	-		-	89.0	11.0	-	
Using long-acting method	-	87.6	12.4	-		-	96.8	3.2	-	
**Emotional response to potential pregnancy**										
Very happy	50.3	22.4	3.3	24.0	0.122	18.7	69.5	5.5	6.2	**0.001**
Happy	25.8	33.7	11.3	29.2		15.0	67.9	5.1	12.0	

Karp C, *et al. BMJ Sex Reprod Health* 2021; 47:252–260. doi: 10.1136/bmjsrh-2020-200944

Mixed feelings	44.2	34.0	5.7	16.2		7.8	76.3	4.8	11.1	
Unhappy	45.2	29.0	10.7	15.2		11.5	68.3	6.0	14.1	
Very unhappy	42.2	24.4	3.9	29.4		12.2	66.6	5.3	16.0	
COVID-19-related										
**Household income loss**										
None	53.8	25.3	6.1	14.9	0.070	9.5	76.1	3.1	11.3	0.463
Partial	37.0	27.2	6.1	29.8		12.5	70.0	5.0	12.6	
Complete	42.6	28.3	5.4	23.8		13.2	66.9	6.0	13.9	
**Concern about future loss of income due to COVID-19**										
No	46.7	26.7	5.2	21.4	0.958	15.1	70.4	0.6	13.9	0.164
Yes	41.2	27.0	5.9	25.9		12.5	69.0	5.5	13.1	
**Food insecurity during COVID-19**										
None	42.0	29.3	7.0	21.7	**<0.001**	11.3	71.1	5.4	12.2	**0.040**
Chronic stable	54.2	6.6	4.0	35.2		18.5	64.0	3.0	14.5	
Increased	31.6	25.2	0.9	42.3		14.3	64.8	5.9	15.1	
**Able to socially distance**										
No	32.1	36.0	8.3	23.7	**0.039**	13.0	70.3	4.7	11.9	0.586
Yes	44.9	23.9	5.2	26.0		12.4	68.5	5.6	13.6	
**Concerned about becoming infected with COVID-19**										
Very concerned	44.9	24.0	5.5	25.7	0.122	11.6	69.8	5.1	13.5	0.270
Concerned	24.8	39.1	4.0	32.2		16.5	65.6	5.2	12.7	
A little/Not concerned	43.8	29.7	12.2	14.4		15.5	67.3	7.5	9.8	

[a]Education in Burkina Faso distinguished women who never went to school (lower) from women who went to school (higher). In Kenya, education distinguished women who have secondary school or higher (higher) from those who have less education (lower) . Row totals are presented. Bolded values indicate p<0.05. All estimates are weighted for the complex survey design.

Supplemental Table S3. Proportion of contraceptive non-users during COVID-19 who report COVID-related reasons for non-use of contraception

**Reason reported**	**Burkina Faso (%)**	**Kenya (%)**
Any COVID-19 related reason	3.8	14.4
Healthcare facility closed	1.0	1.0
Fear of COVID-19 infection at facility	1.9	9.5
Lack of transport to facility/Restrictions on movement	1.5	4.3
Preferred method unavailable	1.2	3.2

Note: Only COVID-19-related reasons reported in table. Totals do not sum to 100% by site; Multiple reported reasons possible.

Karp C, *et al. BMJ Sex Reprod Health* 2021; 47:252–260. doi: 10.1136/bmjsrh-2020-200944
