# Supplementary material for: Contraceptive dynamics during COVID-19 in sub-Saharan Africa: longitudinal evidence from Burkina Faso and Kenya
Source: BMJ Sex Reprod Health. 2021 Feb 12:bmjsrh-2020-200944. doi: 10.1136/bmjsrh-2020-200944 (PMC7886665; doi:10.1136/bmjsrh-2020-200944)
Supplement: Supplementary file 1 [file bmjsrh-2020-200944supp001.pdf]

PMA COVID-19 Survey

Date: 28 Apr 2020
Version: 8

country specific
For questions, email info@pmadata.org

PMA COVID-19 Survey

IDENTIFICATION			
**NO**	**QUESTIONS AND FILTERS**	**CODING CATEGORIES**	**Relevant if:**
001b	**Enter your name below.**<br>*Please record your name*	Interviewer's Name	001a=0
002a	**Current date and time.**<br>[ODK will display on screen]<br>**Is this date and time correct?**	Yes .... 1<br>No 0	Always
002b	**Record the correct date and time.**	**Day** / **Month** / **Year**<br>**Hours** / **Min** / **AM/PM**	002a=0
003a	**LOCATION INFORMATION 1**	LOCATION INFORMATION 1a .... 1<br>LOCATION INFORMATION 1b .... 2<br>LOCATION INFORMATION 1c .... 3<br>LOCATION INFORMATION 1d .... 4<br>LOCATION INFORMATION 1e .... 5<br>LOCATION INFORMATION 1f .... 6<br>LOCATION INFORMATION 1g 7	Always
003b	**LOCATION INFORMATION 2**	ODK will populate a list of appropriate LOCATION INFORMATION 2 based on the LOCATION INFORMATION 1 selected	Always
003c	**LOCATION INFORMATION 3**	ODK will populate a list of appropriate LOCATION INFORMATION 3 based on the LOCATION INFORMATION 2 selected.	Always
003d	**LOCATION INFORMATION 4**	ODK will populate a list of appropriate LOCATION INFORMATION 4 based on the LOCATION INFORMATION 3 selected	Always
004	**Enumeration area**	ODK will populate a list of appropriate enumeration areas based on the LOCATION INFORMATION 4 selected	Always

Karp C, *et al. BMJ Sex Reprod Health* 2021;0:1–9. doi: 10.1136/bmjsrh-2020-200944

PMA COVID-19 Survey

PERFORMANCE MONITORING FOR ACTION

NO	QUESTIONS AND FILTERS	CODING CATEGORIES	Relevant if:
008	**Call attempt number**	Call attempt number	Always
009	**Did someone answer your call?**	Yes .... 1<br>No 0	Always
010	**Hello. My name is ____________ calling from the [PARTNER ORGANIZATION]. May I speak to [RESPONDENT'S NAME]?**	Yes .... 1<br>No 0	009 = 1
011	**Do you have the correct person on the phone?**	Yes .... 1<br>No 0	010 = 1
012	**Record the result of the phone call.**	Reached correct participant .... 1<br>No answer .... 2<br>Wrong number .... 3<br>Phone switched off .... 4<br>Phone no longer working .... 5<br>Participant not available 6	Always

INFORMED CONSENT

Read the greeting on the next screen:

NO	QUESTIONS AND FILTERS	CODING CATEGORIES	Relevant if:
013	*Read the verbal consent text.*<br><br>*Then, ask:*<br>**May I begin the interview now?**	Yes .... 1<br>No 0	012 = 1
014	**Please tell me your name.**	Respondent's name	013 = 1
015	**Please tell me your age in years.**<br><br>*Enter -88 for do not know*<br>*Enter -99 for no response.*	Age in years	013 = 1
016	**What is the highest level of school you attended?**<br><br>*Only record formal schooling. Do not record bible or koranic school or short courses.*	Never Attended .... 0<br>LEVEL 1 .... 1<br>LEVEL 2 .... 2<br>LEVEL 3 .... 3<br>LEVEL 4 .... 4<br>LEVEL 5 .... 5<br>No response .... -99	013 = 1

Karp C, et al. BMJ Sex Reprod Health 2021;0:1–9. doi: 10.1136/bmjsrh-2020-200944

PMA COVID-19 Survey

SECTION 1: BACKGROUND<br>*I would like to ask you some questions about your background and residence*			
**NO**	**QUESTIONS AND FILTERS**	**CODING CATEGORIES**	**Relevant if:**
101	**Are you currently in [PROVINCE/REGION/COUNTY]?**	Yes ....1<br>No ....0<br>No response ....-99	013 = 1
102	**In which [PROVINCE/REGION/COUNTY] are you staying now?**	LOCATION INFORMATION 1a....................1<br>LOCATION INFORMATION 1b ....2<br>LOCATION INFORMATION 1c ....3<br>LOCATION INFORMATION 1d ....4<br>LOCATION INFORMATION 1e ....5<br>LOCATION INFORMATION 1f ....6<br>LOCATION INFORMATION 1g 7	101 = 0
103	**Do you have a dedicated place to wash your hands at your current residence?**	Yes ....1<br>No ....0<br>No response ....-99	013 = 1
104	**Do you have soap at this place to wash your hands?**	Yes ....1<br>No ....0<br>No response ....-99	103 = 1
105	**How many times did you wash your hands with soap yesterday?**	Zero (0)....................1<br>1 to 3....................2<br>4 to 10....................3<br>More than 10....................4<br>No response ....-99	103 = 1

PMA COVID-19 Survey

PMA PERFORMANCE MONITORING FOR ACTION

SECTION 2: AWARENESS OF COVID-19, and MEDIA EXPOSURE

The next series of questions are about COVID-19, also called Coronavirus.

NO	QUESTIONS AND FILTERS	CODING CATEGORIES	Relevant if:
201	**How much, if anything, have you heard or read about the recent Coronavirus (COVID-19) outbreak?**<br><br>*Read all options*	A lot ....1<br>Some ....2<br>A little ....3<br>Not at all....4<br>No response ....-99	013 = 1
202	**How did you learn about Coronavirus (COVID-19)?**<br><br>*Do not read responses.*<br>*Select all that apply*	Newspaper....1/0<br>Radio ....1/0<br>Television....1/0<br>Poster / billboard ....1/0<br>Town crier ....1/0<br>Phone message ....1/0<br>Family ....1/0<br>Friends / neighbors ....1/0<br>Community/religious leaders....1/0<br>Social media (Twitter, Facebook, WhatsApp) ....1/0<br>Health personnel....1/0<br>Messages from government or authorities ....1/0<br>School....1/0<br>None of these sources ....-99	201 ≠ 4
203	**Have you ever heard of an emergency number or call center for reporting suspected cases of Coronavirus (COVID-19)?**<br><br>**Probe: If yes, ask if the respondent knows the number.**	Yes, knows the number ....1<br>Yes, but does not know the number....2<br>No ....3<br>No response ....-99	013 = 1
204	**Which of these sources do you trust for accurate information about Coronavirus (COVID-19)?**<br><br>*Read all options*<br>*Select all that apply*	Newspaper....1/0<br>Radio ....1/0<br>Television....1/0<br>Poster / billboard ....1/0<br>Town crier ....1/0<br>Phone message ....1/0<br>Family ....1/0<br>Friends / neighbors ....1/0<br>Community/religious leaders....1/0<br>Social media (Twitter, Facebook, WhatsApp) ....1/0<br>Health personnel....1/0<br>Messages from government or authorities ....1/0<br>School....1/0<br>None of these sources ....-99	201 ≠ 4
205	**Do you trust the call center number for accurate information?**	Yes ....1<br>No ....0<br>No response ....-99	203 = 1 or 2
206	**Have you tried to call the emergency number or call center?**	Yes ....1<br>No ....0<br>No response ....-99	013 = 1

Karp C, *et al. BMJ Sex Reprod Health* 2021;0:1–9. doi: 10.1136/bmjsrh-2020-200944

PMA COVID-19 Survey

SECTION 3: COVID-19 RISK PERCEPTION

Now I would like to ask about your views about Coronavirus (COVID-19)*. Your answers will be kept completely confidential.*

NO	QUESTIONS AND FILTERS	CODING CATEGORIES	Relevant if:
301	**If someone in your family caught Coronavirus (COVID-19), would you want to keep it a secret?**	Yes ....1<br>No ....0<br>No response ....-99	201 ≠ 4
302	**How concerned are you about the spread of Coronavirus (COVID-19) in your community?**<br><br>*Read all options*	Very concerned ....1<br>Concerned ....2<br>A little concerned ....3<br>Not concerned ....4<br>No response ....-99	201 ≠ 4
303	**Approximately how many people in your community do you think are or have been infected with Coronavirus (COVID-19)?**<br><br>*Read all options*	Most people ....1<br>Some people ....2<br>Few people ....3<br>No one is infected ....4<br>Don't know ....-88<br>No response ....-99	201 ≠ 4
304	**Have any of your close relatives or friends had or have Coronavirus (COVID-19)?**	Yes ....1<br>No ....0<br>Don't know ....-88<br>No response ....-99	201 ≠ 4
305	**How concerned are you about getting infected yourself?**<br><br>*Read all options*	Very concerned ....1<br>Concerned ....2<br>A little concerned ....3<br>Not concerned ....4<br>I am currently / was infected with COVID-19 ....5<br>No response ....-99	201 ≠ 4

PMA COVID-19 Survey

SECTION 4: COVID-19 SYMPTOMS AND TRANSMISSION

Now I would like to ask about your knowledge of COVID-19 symptoms and transmission.

NO	QUESTIONS AND FILTERS	CODING CATEGORIES	Relevant if:
401	**What are common symptoms of Coronavirus (COVID-19) infection?**<br><br>*Do not read responses.*<br>*Select all that apply*	Fever .... 1/0<br>Cough .... 1/0<br>Shortness of breath/difficulty breathing .... 1/0<br>Chest pain........ 1/0<br>Sore throat .... 1/0<br>Runny or stuffy nose .... 1/0<br>Muscle or body aches .... 1/0<br>Headaches........ 1/0<br>Fatigue (tiredness) .... 1/0<br>Diarrhea .... 1/0<br>Loss of taste .... 1/0<br>Loss of smell .... 1/0<br>Rash .... 1/0<br>Dizziness .... 1/0<br>Sneezing........ 1/0<br>Other........ 1/0<br>Do not know any symptoms .... 1/0<br>No response ....-99	201 ≠ 4
402	**Which of the following statements are true about the Coronavirus (COVID-19) infection?**<br><br>*Read all options*	Some people cannot be infected with Coronavirus (COVID-19) ....1/0/-88/-99<br>Most people experience mild or no symptoms ....1/0/-88/-99<br>Most people develop serious illness requiring hospitalization ....1/0/-88/-99<br>People can be infected and not have symptoms........1/0/-88/-99<br>Only people with symptoms are contagious ....1/0/-88/-99<br>You can become infected by shaking hands with someone who is infected ....1/0/-88/-99<br>You can become infected by close contact with infected people even if you are not touching ....1/0/-88/-99<br>People of all ages can become infected ....1/0/-88/-99<br>Coronavirus (COVID-19) is mostly a risk to rich people........1/0/-88/-99	201 ≠ 4

PMA COVID-19 Survey

NO	QUESTIONS AND FILTERS	CODING CATEGORIES	Relevant if:
403	**Which of the following actions can reduce the risk of being infected?**<br><br>*Read all options*	Washing hands with soap and water frequently ....1/0/-88/-99<br>Washing hands with hand sanitizer frequently ....1/0/-88/-99<br>Avoiding any close contact (2 meters) with people when you go out......1/0/-88/-99<br>Staying in your home ....1/0/-88/-99<br>Getting vaccinated ....1/0/-88/-99<br>Traditional practices........1/0/-88/-99<br>Wearing something that covers your mouth and nose when you go out (a mask)........1/0/-88/-99<br>Avoiding shaking hands with others ....1/0/-88/-99<br>Coughing/sneezing into your elbow or tissue........1/0/-88/-99<br>Prayer 1/0/-88/-99	201 ≠ 4
404	**Have you taken any action to prevent becoming infected?**	Yes ....1<br>No ....0<br>No response ....-99	201 ≠ 4
405	**What have you done?**<br><br>*Read all options*<br>*Select all that apply*	Washing hands with soap and water frequently ....1/0<br>Washing hands with hand sanitizer frequently ....1/0<br>Avoiding any close contact (2 meters) with people when you go out........1/0<br>Staying in your home ....1/0<br>Getting vaccinated ....1/0<br>Traditional practices........1/0<br>Wearing something that covers your mouth and nose when you go out (a mask)........1/0<br>Avoiding shaking hands with others ....1/0<br>Coughing/sneezing into your elbow or tissue........1/0<br>Prayer ....1/0<br>Other........1/0<br>No response ....-99	404 = 1
406	**Are you able to avoid contact with people outside of your household?**	Yes ....1<br>No ....0<br>No response ....-99	201 ≠ 4
407	**What are some reasons why you might not be able to avoid contact with people outside of your household?**<br><br>*Read all options*<br>*Select all that apply*	My work or way of earning money requires me to leave the house........1/0<br>I need to visit the market........1/0<br>I need to visit the water source / well ....1/0<br>My studies require me to leave the household ....1/0<br>I need to attend funerals in the community ....1/0<br>I need to attend religious services........1/0<br>I need to visit my family/relatives........1/0<br>To seek out health care ....1/0<br>No response ....-99	406≠ 1

PMA COVID-19 Survey

SECTION 5: SOCIAL CONSEQUENCES OF COVID-19

We understand that COVID-19 is affecting many people's lives. We're interested in learning more about how you are being affected.

NO	QUESTIONS AND FILTERS	CODING CATEGORIES	Relevant if:
501	**Since the Coronavirus (COVID-19) restrictions began, how much of a loss of income has your household experienced?**<br><br>*Read all options*	None ....1<br>Complete ....2<br>Partial ....3<br>No response ....-99	013 = 1
502	**Since the Coronavirus (COVID-19) restrictions began, how much of a loss of income have you experienced?**<br><br>*Read all options*	Large ....1<br>Moderate....2<br>Small....3<br>Has no income....4<br>No response ....-99	501 = 3
503	**Are you worried about the impact of Coronavirus (COVID-19) on your household's finances in the future?**	Yes ....1<br>No....0<br>Don't know....-88<br>No response ....-99	201 ≠ 4
504	**Since the Coronavirus (COVID-19) restrictions began, did you or any household member go a whole day and night without eating anything because there was not enough food?**	Yes ....1<br>No....0<br>Don't know....-88<br>No response ....-99	201 ≠ 4
505	**Is this more common now compared to before the Coronavirus (COVID-19) restrictions began?**	Yes ....1<br>No....0<br>Don't know....-88<br>No response ....-99	504 = 1
506	**Since the Coronavirus (COVID-19) restrictions began, how often has this happened?**	Rarely (1-2 times) ....1<br>Sometimes (3-10 times) ....2<br>Often (more than 10 times) ....3<br>Don't know....-88<br>No response ....-99	504 = 1
507	**Are you currently married or living together with a man as if married?**<br><br>**Probe: If no, ask whether the respondent is divorced, separated, or widowed.**	Yes, currently married....1<br>Yes, living with a man ....2<br>Not currently in union:<br>Divorced / separated....3<br>Not currently in union:<br>Widow....4<br>No, never in union....5<br>No response ....-99	013 = 1
508	**Since the Coronavirus (COVID-19) restrictions began, who usually makes decisions about making household purchases for daily needs: you, your husband/partner, you and your husband/partner jointly, or someone else?**	Respondent ....1<br>Husband/partner ....2<br>Respondent and husband/partner....3<br>Someone else....4<br>No response ....-99	507 = 1 or 2
509	**Are you currently economically reliant on your husband/partner for basic needs?**	Yes ....1<br>No....0<br>No response ....-99	507 = 1 or 2
510	**Are you more economically reliant on your husband/partner now than before the Coronavirus (COVID-19) restrictions began?**	Yes ....1<br>No....0<br>Don't know....-88<br>No response ....-99	509 = 1

PMA COVID-19 Survey

PMA PERFORMANCE MONITORING FOR ACTION

SECTION 6: COVID-19 AND FAMILY PLANNING

Now I would like to ask you about pregnancy and family planning.

NO	QUESTIONS AND FILTERS	CODING CATEGORIES	Relevant if:
601	**Are you currently pregnant?**	Yes ....1<br>No ....0<br>Unsure .... 2<br>No response ....-99	013 = 1
602	**Have you needed to visit a health facility since the Coronavirus (COVID-19) restrictions began, including for family planning?**	Yes ....1<br>No ....0<br>No response ....-99	013 = 1
603	**What was the reason why you needed to visit a health facility?**<br><br>*Read all options*<br>*Select all that apply*	Family planning services ....1/0<br>ANC ....1/0<br>Delivery ....1/0<br>PNC ....1/0<br>Child's health ....1/0<br>Immunization ....1/0<br>Pick up of regular medications ....1/0<br>HIV....1/0<br>Emergency services....1/0<br>General health services ....1/0<br>Other....1/0<br>No response ....-99	602 = 1
604	**Have you experienced any of the following difficulties in accessing healthcare services since the Coronavirus (COVID-19) restrictions began?**<br><br>*Read all options*<br>*Select all that apply*	Healthcare facility or doctor's office closed, appointment not possible ....1/0<br>Partner does not approve....1/0<br>No transportation to access healthcare services ....1/0<br>Unable to access services because of government restrictions on movement ...1/0<br>Unable to afford healthcare services....1/0<br>Fear of being infected with COVID-19 at healthcare facilities ....1/0<br>No response ....-99	013 = 1
605	**Did you successfully access the health services you needed?**	Yes ....1<br>No ....0<br>No response ....-99	602 = 1
606a	**Now I have some questions about the future. Would you like to have a child or would you prefer not to have any children?**	Have a/another child ....1<br>No more/prefer no children ....2<br>Says she can't get pregnant....3<br>Undecided / Don't know ....-88<br>No response ....-99	013 = 1<br>AND<br>601 ≠ 1
606b	**Now I have some questions about the future. After the child you are expecting now, would you like to have another child, or would you prefer not to have any more children?**	Have a/another child ....1<br>No more/prefer no children ....2<br>Says she can't get pregnant....3<br>Undecided / Don't know ....-88<br>No response ....-99	601 = 1

9

PMA COVID-19 Survey

NO	QUESTIONS AND FILTERS	CODING CATEGORIES	Relevant if:
607a	**How long would you like to wait from now before the birth of a child?**<br><br>*If you select months or years, you will enter a number for x on the next screen.*<br><br>*Select "Years" if more than 36 months.*<br><br>*Please check that you correctly entered the value for months/years.*	Months (1) [ ]<br>Years (2) [ ]<br>Soon / now ....3<br>Says she can't get pregnant....4<br>Other....5<br>Don't know....-88<br>No response ....-99	606a = 1
607b	**After the birth of the child you are expecting now, how long would you like to wait before the birth of another child?**<br><br>*If you select months or years, you will enter a number for x on the next screen.*<br><br>*Select "Years" if more than 36 months.*<br><br>*Please check that you correctly entered the value for months/years.*	Months (1) [ ]<br>Years (2) [ ]<br>Soon / now ....3<br>Says she can't get pregnant....4<br>Other....5<br>Don't know....-88<br>No response ....-99	606b = 1
608	**Have you changed your mind about wanting to get pregnant due to concerns about Coronavirus (COVID-19)?**	Yes ....1<br>No....0<br>No response ....-99	013 = 1<br>AND<br>601 ≠ 1<br>AND<br>201 ≠ 4
609	**If you got pregnant now, how would you feel?**<br><br>*Read all options*	Very happy.... 1<br>Sort of happy .... 2<br>Mixed happy and unhappy.... 3<br>Sort of unhappy .... 4<br>Very unhappy.... 5<br>No response ....-99	013 = 1<br>AND<br>601 ≠ 1
610	**Are you or your partner currently doing something or using any method to delay or avoid getting pregnant?**	Yes ....1<br>No....0<br>No response ....-99	013 = 1<br>AND<br>601 ≠ 1
611	**You said that you do not want any more children and that you are not using a method to avoid pregnancy. Can you tell me the reason why you are not using a method to prevent pregnancy?**<br><br>*Read all options*<br>*Select all that apply*	Healthcare facility or doctor's office closed, appointment not possible....1/0<br>Partner does not approve....1/0<br>No transportation to access services ....1/0<br>Preferred contraceptive method unavailable....1/0<br>Unable to access services because of government restrictions on movement ....1/0<br>Unable to afford FP services....1/0<br>Fear of being infected with COVID-19 at healthcare facilities ....1/0<br>Other....1/0<br>No response ....-99	(013 = 1<br>AND<br>610 ≠ 1)<br><br>AND<br><br>(606a = 2<br>or<br>606b = 2)

PMA COVID-19 Survey

NO	QUESTIONS AND FILTERS	CODING CATEGORIES	Relevant if:
612	**What method are you using?**<br><br>**Probe: Anything else?**<br><br>*Select all methods mentioned. Be sure to scroll to bottom to see all choices.*	Female sterilization .... 1/0<br>Male sterilization .... 1/0<br>Implant .... 1/0<br>IUD .... 1/0<br>Injectables .... 1/0<br>Pill .... 1/0<br>Emergency Contraception .... 1/0<br>Male Condom .... 1/0<br>Female Condom .... 1/0<br>Diaphragm .... 1/0<br>Foam/Jelly .... 1/0<br>Std. Days/Cycle beads .... 1/0<br>LAM .... 1/0<br>Rhythm method .... 1/0<br>Withdrawal .... 1/0<br>Other traditional methods .... 1/0<br>No response .... -99	610 = 1
613	**Have you used emergency contraception since the Coronavirus (COVID-19) restrictions began?**	Yes .... 1<br>No .... 0<br>No response .... -99	013 = 1<br>AND<br>601 ≠ 1<br>AND<br>612 ≠ EC

PMA COVID-19 Survey

PMA PERFORMANCE MONITORING FOR ACTION

SECTION 7: FOLLOW-UP CONSENT			
701	**Thank you for the time you have kindly granted us. We look forward to speaking with you again.**<br><br>**Do you have a better number we can reach you at in case we would like to follow-up with you in the future?**	Yes....1<br>No....0<br>No response....-99	013 = 1
702	**What is the updated number?**<br><br>*Enter an #-digit number without the country code. Do not include spaces or dashes. Enter 0 for no response.*<br><br>[UPDATE TO MATCH COUNTRY PHONE SYSTEM]	Phone number:	701 = 1
703	**Can you repeat the number again?**<br><br>*Enter an #-digit number without the country code. Do not include spaces or dashes. Enter 0 for no response.*<br><br>[UPDATE TO MATCH COUNTRY PHONE SYSTEM]<br><br>*Number entered must match previously entered number.*	Phone number:	702 ≠ 0

QUESTIONNAIRE RESULT			
**Thank the respondent for her time.**<br>*The respondent is finished, but there are still more questions for you to complete.*			
**NO**	**QUESTIONS AND FILTERS**	**CODING CATEGORIES**	**Relevant if:**
098	**In what language was this interview conducted?**	English....1<br>French....2<br>Language 3....3<br>Language 4....4<br>Language 5....5<br>Language 6....6<br>Other....96	013 = 1
099	**Record the result of the COVID-19 Questionnaire.**	Completed....1<br>Postponed....2<br>Refused....3<br>Partly completed....4<br>Other....5	Always
